# Meta-analysis of synovial fluid polymerase chain reaction for diagnosing periprosthetic hip and knee infection

**DOI:** 10.1186/s13018-021-02813-8

**Published:** 2022-01-04

**Authors:** Cheng Li, Hao Li, Xue Yang, Fang-Zheng Zhu, Chi Xu, Andrej Trampuz

**Affiliations:** 1 Department of Orthopaedic Surgery, Beijing Jishuitan Hospital, Beijing, China; 2 Center for Musculoskeletal Surgery (CMSC), Charité–Universitätsmedizin Berlin, Berlin, Germany; 3 Department of Orthopaedic Surgery, General Hospital of People’s Liberation Army, Beijing, China

**Keywords:** Meta-analysis, Diagnosis, Arthroplasty, Periprosthetic joint infection, Synovial fluid, Polymerase chain reaction

## Abstract

**Background and objective:**

The purpose of this study was to estimate the diagnostic performance of synovial fluid polymerase chain reaction (PCR) in periprosthetic hip and knee infection, and whether synovial fluid PCR has greater diagnostic significance than conventional methods.

**Methods:**

The literature databases PubMed, Scopus, and the Web of Science were searched for English articles describing periprosthetic joint infection (PJI) diagnosis by synovial fluid PCR. Articles were limited to the period between January 1990 and December 2019. Subsequently, conventional methods that were used on at least two occasions were included for further analysis. Data analysis was performed using the Meta-DiSc and Stata software.

**Results:**

Eleven studies with 1360 cases were included in the meta-analysis. The pooled sensitivity, specificity, and diagnostic odds ratio (DOR) of synovial fluid PCR were 0.70 (95% CI 0.66–0.74), 0.92 (95% CI 0.90–0.93), and 37.4 (95% CI 17.77–78.74), respectively.

**Conclusions:**

Synovial fluid PCR provides an effective tool for rapid diagnosis of PJI, and also in the early stages of culture-negative bacterial infections.

## Background

Periprosthetic joint infection (PJI) is one of the most severe complication following hip or knee arthroplasty, with high morbidity, mortality and costs [1–5]. However, its diagnosis remains a challenge in the management of PJI. To date, there is no test available that rapidly and accurately diagnoses infection with a sensitivity and specificity of 100% [6]. A mixture of several diagnostic methods or diagnostic definition that guide the diagnosis of PJI is currently the most common approach used [7].

Joint aspiration is an invasive diagnostic method and often used as the first step in suspected PJI cases. Currently, various synovial fluid tests have been applied in the clinical diagnosis of PJI [8, 9]. Non-microbiological analysis of the synovial fluid white cell count and polymorphonuclear (PMN) leukocytes, alpha-defensin, leukocyte esterase (LE), and C-reactive protein (CRP) were included in the new definition of PJI from the Musculoskeletal Infection Society (MSIS) guideline of 2018 [10]. Although these tests are of high diagnostic value for PJI cases, they are unable to identify the causative pathogen. Conventional synovial fluid culture still appears to be an irreplaceable diagnostic approach for the detection of microorganisms. In recent years, polymerase chain reaction (PCR) was also used in the diagnosis of PJI. A number of reports found that synovial fluid PCR could rapidly detect rare bacterial infections, which is in contrast to conventional synovial fluid culture [11, 12]. However, it remains unknown whether better diagnostic results are observed using PCR compared to tissue culture, with several conclusions arguable [13, 14]. Although a meta-analysis of PCR for diagnosis of PJI has been previously performed using results combined from synovial fluid, periprosthetic tissue, and sonicate fluid samples [15], the diagnostic value of synovial fluid PCR in the periprosthetic hip and knee remains unclear.

The aim of the present meta-analysis was to assess the diagnostic accuracy of synovial fluid PCR in periprosthetic hip and knee infection. Furthermore, we examined whether synovial fluid PCR has a better diagnostic value compared to conventional methods, especially synovial fluid culture.

## Methods

### Search strategy

A systematic literature search of the electronic databases of Web of Science, PubMed, and Scopus was performed for manuscripts of English language between January 1990 and December 2019. The following medical subject headings (MeSH) or text keywords were used: “arthroplasty or joint prosthesis or joint replacement or periprosthetic joint or prosthetic joint”, “infection or infectious or infected”, “synovial fluid”, and “PCR or polymerase chain reaction”.

Two authors (LC and LH) independently selected research papers according to the following inclusion criteria: (1) human studies related to synovial fluid PCR in periprosthetic hip or knee infection; (2) clear description of the diagnosis standard of PJI; and (3) values on true-positive (TP), false-positive (FP), true-negative (TN), and false-negative (FN) were provided or could be computed.

The combined database was imported to EndNote X7 (Thomson Reuters, New York, NY, USA, 2013). All relevant publications on the synovial fluid PCR method in detecting periprosthetic hip or knee joint infection and their reference list were reviewed. Furthermore, included studies from two previous meta-analyses of synovial PCR were reviewed [15, 16]. In addition, other diagnostic methods that appeared accumulatively on at least two occasions in all studies were collected. Diagnostic classification values of TP, FP, TN, and FN were included and further compared with synovial fluid PCR.

### Data extraction and study quality assessment

Characteristics of the included studies were collected by two reviewers independently and assessed subsequently by a third reviewer. The following information was extracted: first author, year of publication, country, study design, number of total cases, infection site, acquisition time, diagnostic criteria, type of PCR, target gene, antimicrobial use before specimen collection, and diagnostic sensitivity and specificity. The quality of all identified synovial fluid studies was evaluated using the Quality Assessment of Diagnostic Accuracy Studies (QUADAS-2) guidelines.

### Statistical analysis

To estimate the diagnostic value of synovial fluid PCR for PJI detection, all statistical analyses were performed using Meta-DiSc (version 1.4, Unit of Clinical Biostatistics team, Madrid, Spain) and Stata software (version 14.0, StataCorp, College Station, TX, USA).* I*^2^ was calculated to evaluate heterogeneity among the studies. If *I*^2^ > 50%, the random-effects model was used. Meta-regression analyses were performed to further assess the potential source of heterogeneity, such as type of prosthesis, number of patients, acquisition time, antibiotic use, sample condition, diagnostic standard, and target gene. Deeks’ funnel plot asymmetry test was used to evaluate potential publication bias.

## Results

### Search results

**Fig. 1:**
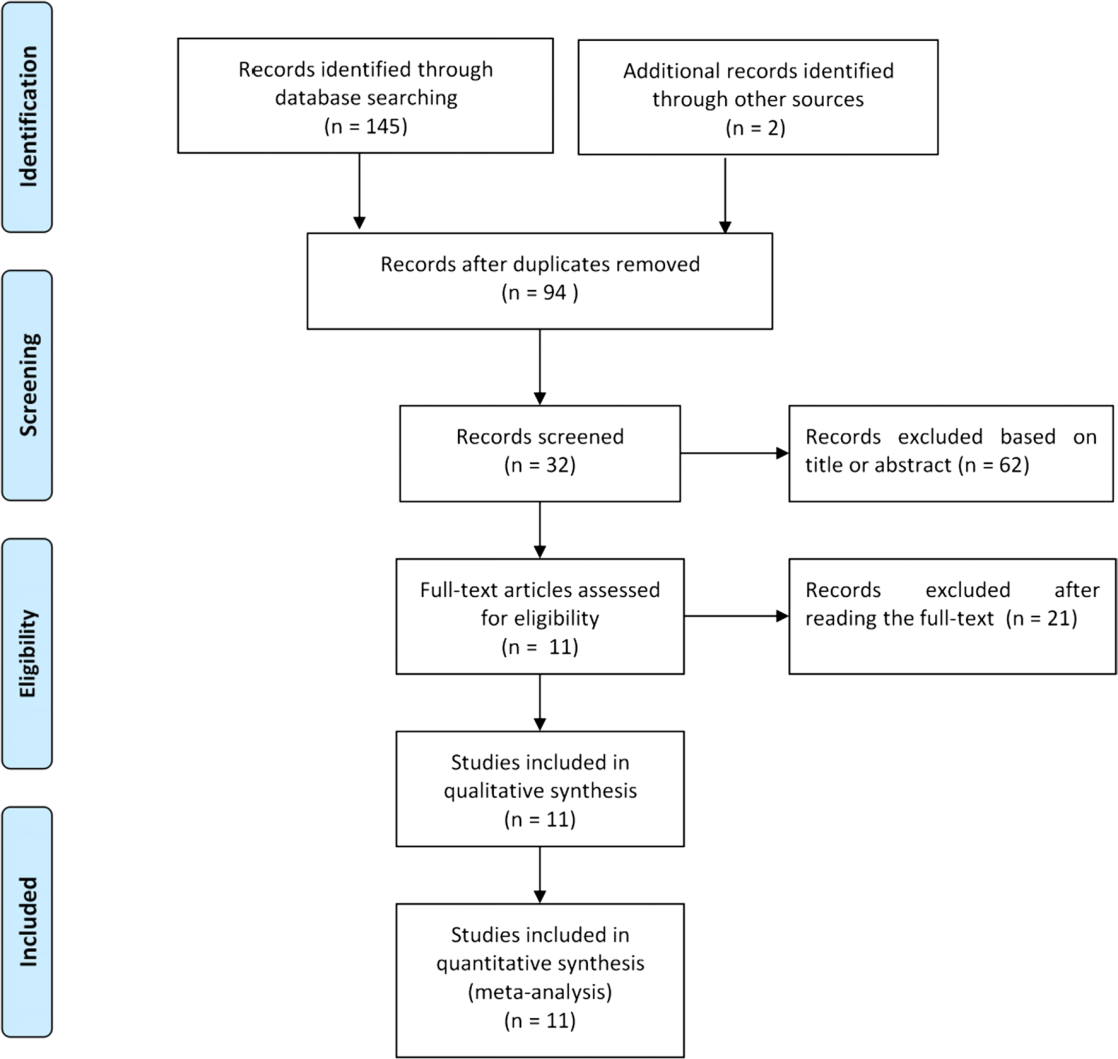
Flow diagram of the selection process for eligible studies

**Fig. 2:**
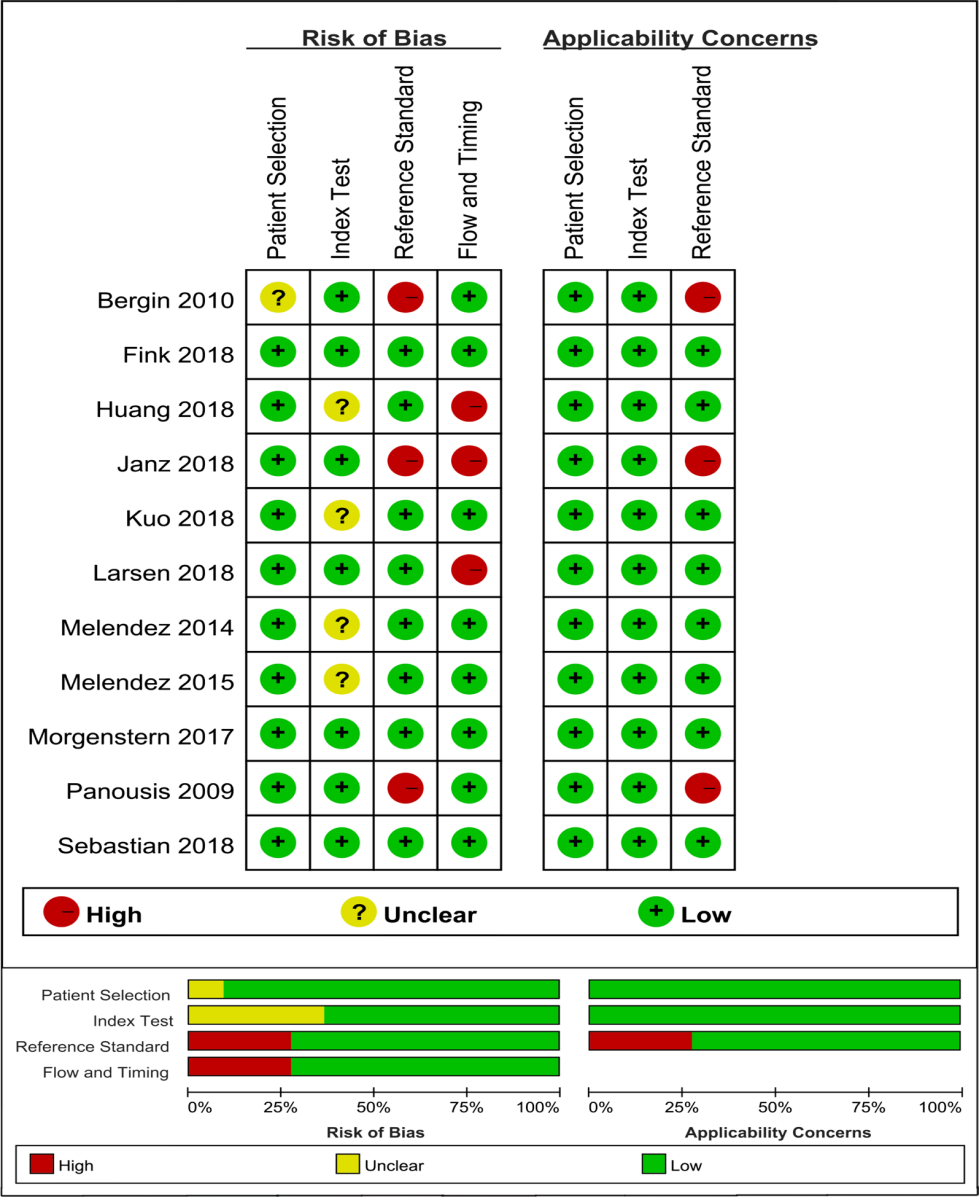
Methodological quality assessment of the included studies. CI, confidence interval; AUC, Area under the curve; DOR, Diagnostic odds ratio; CRP, C-reactive protein; IL-6, Interleukin-6; LE, Leukocyte esterase; PCR, Polymerase chain reaction; PMN, Polymorphonuclear leukocytes; WBC, White blood cell

Of the identified 145 primary articles, two records were extracted from the reference list of synovial fluid PCR-related studies [17, 18]. Fifty-one studies were excluded due to duplication reasons. A further 83 were excluded after further reviewing the title, abstract, and full text. Finally, a total of 11 studies were considered suitable for meta-analysis (Fig. 1) [13, 14, 17–25]. Characteristics of the studies included are summarized in Table 1. Among these studies, five diagnostic methods could be compared with synovial fluid PCR. The QUADAS-2 quality assessments for the included studies are shown in Fig. 2.

**Table 1:** Characteristics of included studies in the meta-analysis

References	Years	Study design	No. of cases	Location	Acquisition time	PCR type	Target gene	Diagnostic standard	Sensitivity (%)	Specificity (%)	Antibiotic treatment
[19]	2010	Prospective	64	Knee	Preoperative	RT-qPCR	16S rRNA	M, H	71	100	Y
[14]	2018	Prospective	116	Knee	Preoperative	PCR SEQ	16S/18S rRNA	MSIS	55.60	82	N
[20]	2018	Prospective	114	Hip, knee	Preoperative	PCR SEQ	16S rRNA	MSIS	71.40	98.50	Y
[21]	2016	Retrospective	284	Knee	Preoperative	PCR panel	Home designed primers	MSIS	55.60	91.80	Y
	2016	Retrospective	62	Knee	Preoperative	PCR-ESI/MS	Specific primer	MSIS	82.30	84.40	Y
[22]	2018	Prospective	87	Hip, knee	Intraoperative	PCR SEQ	16S rRNA	MSIS	68.10	100	NA
[23]	2018	Prospective	17	Hip, knee	Preoperative	PCR lateral flow immunoassay	16S rDNA (multiplex)	P, H, M	87.50	100	NA
	2018	Prospective	60	Hip, knee	Preoperative	PCR lateral flow immunoassay	16S rDNA (multiplex)	P, H, M	82	89	NA
[25]	2018	Prospective	142	Hip, knee	Preoperative	Multiplex PCR	Specific primer	EBJIS	60	89	N
[24]	2005	Prospective	92	Hip, knee	Intraoperative	Broad-range PCR	Specific primer	P, M, R	92	74	N
[13]	2018	Prospective	214	Hip, knee	Preoperative	RT-PCR	16S/28S rRNA	ICM	100	99.50	N
[18]	2014	Retrospective	103	Knee	NA	PCR-ESI/MS	Specific primer	MSIS	81	95	Y
[17]	2018	Retrospective	67	Hip, knee	NA	RT BR-PCR	16S rRNA	MSIS	83	85.70	N

BR, broad-range; C, clinical signs of infection; EBJIS, European Bone and Joint Infection Society; ESI/MS, Electrospray ionization mass spectrometry; H, histological examination; ICM, International Consensus Meeting; MSIS, Musculoskeletal Infection Society; M, microbiological or laboratory examination; NA, not available; N, no; P, presence sinus tract or purulence around the prosthesis; PCR, polymerase chain reaction; qPCR, quantitative PCR; R, radiographic images; RT, real-time; SEN, sensitivity; SPE, specificity; Y, yes

### Diagnostic accuracy

**Fig. 3:**
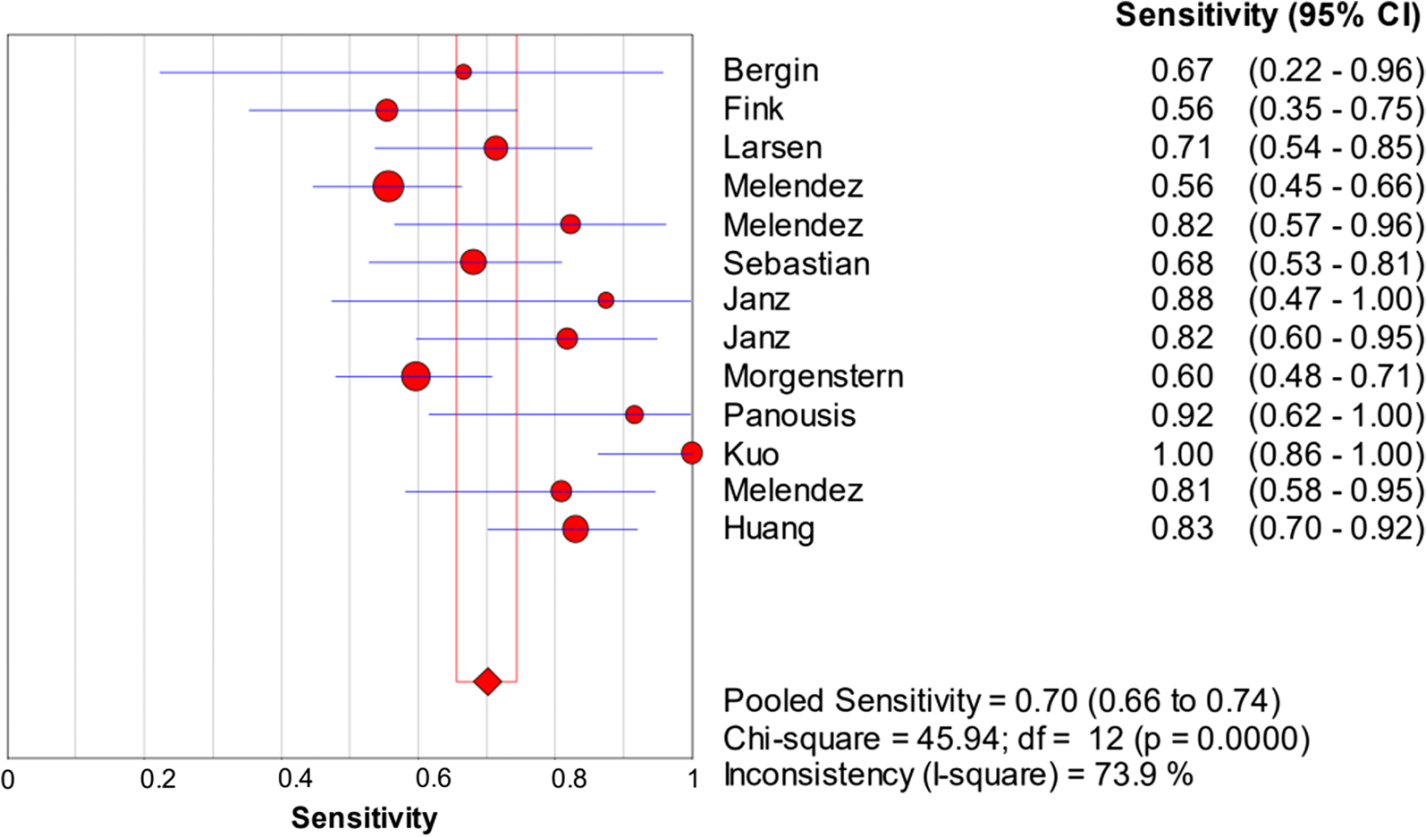
Forest plots of sensitivity of synovial fluid PCR for PJI diagnosis

**Fig. 4:**
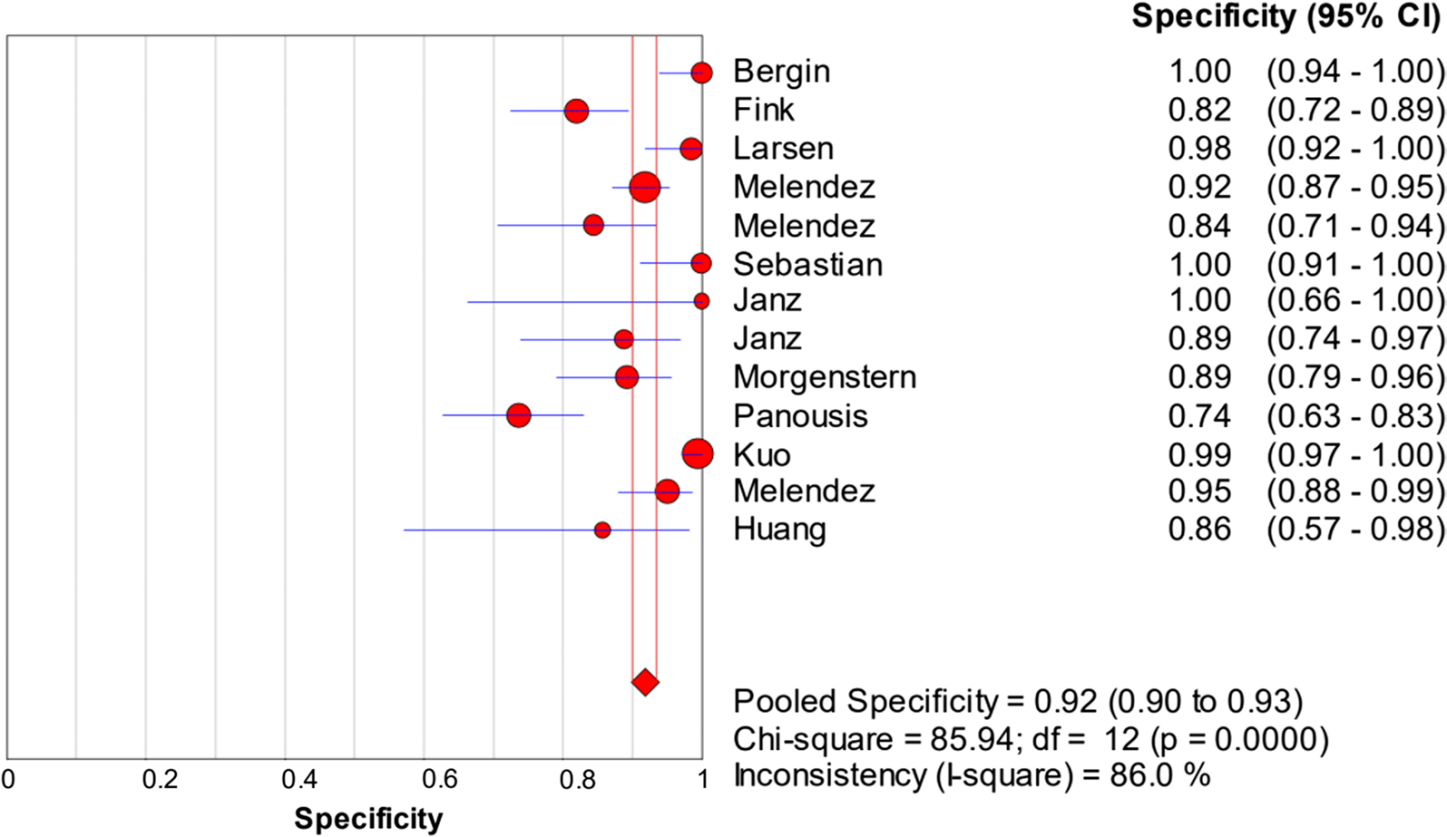
Forest plots of specificity of synovial fluid PCR for PJI diagnosis

**Fig. 5:**
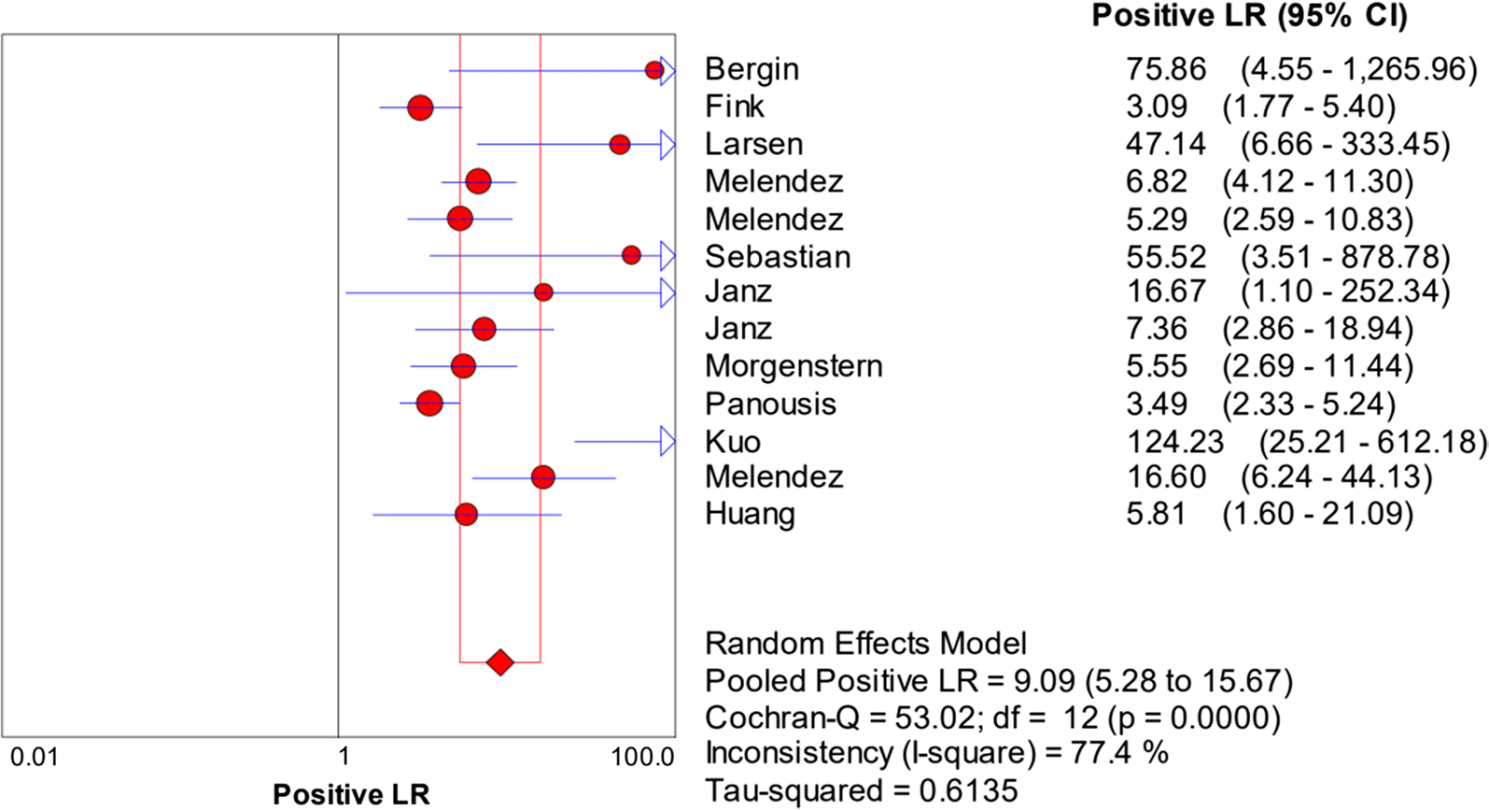
Forest plots of positive likelihood ratio of synovial fluid PCR for PJI diagnosis

**Fig. 6:**
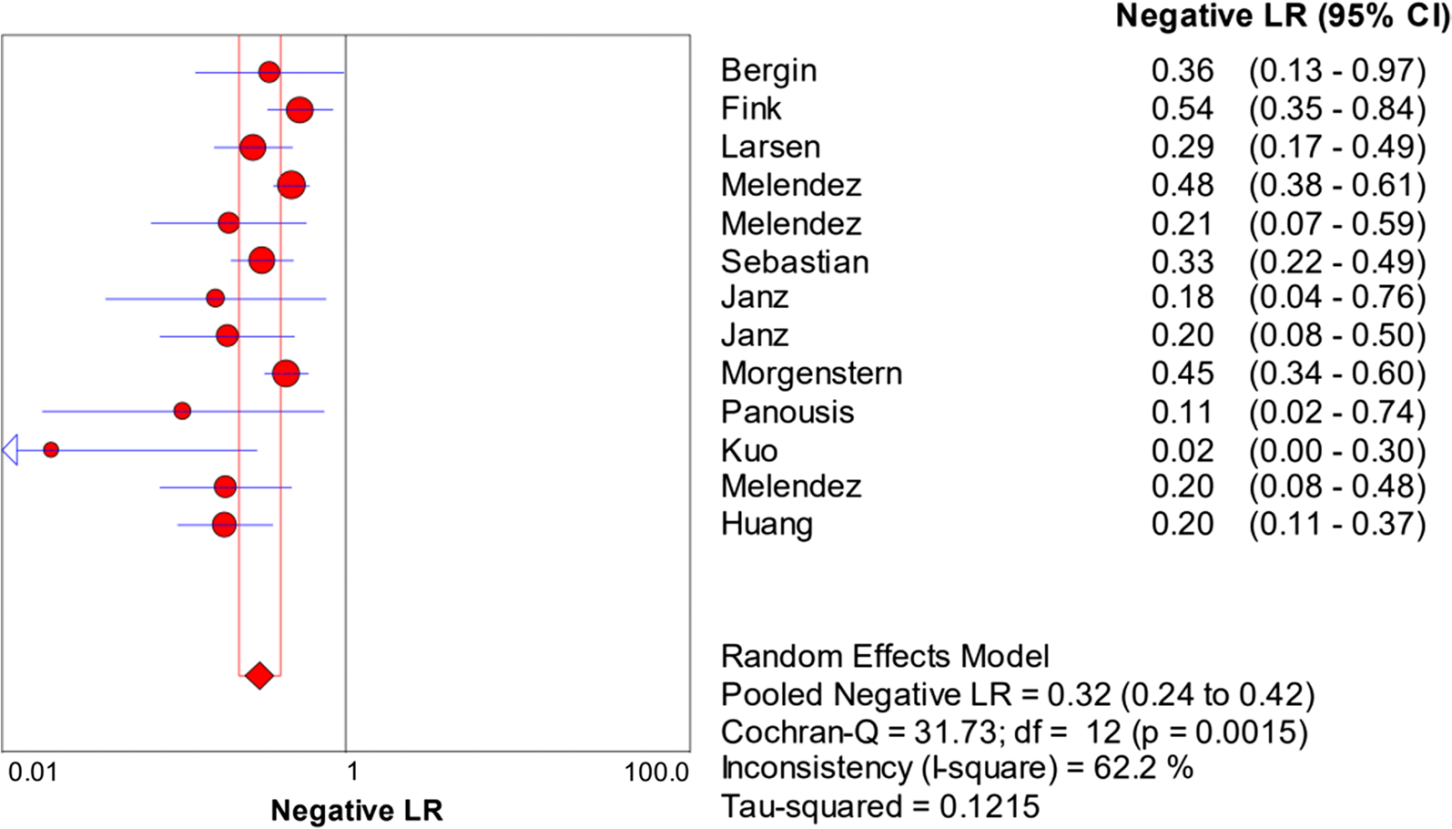
Forest plots of negative likelihood ratio of synovial fluid PCR for PJI diagnosis

**Fig. 7:**
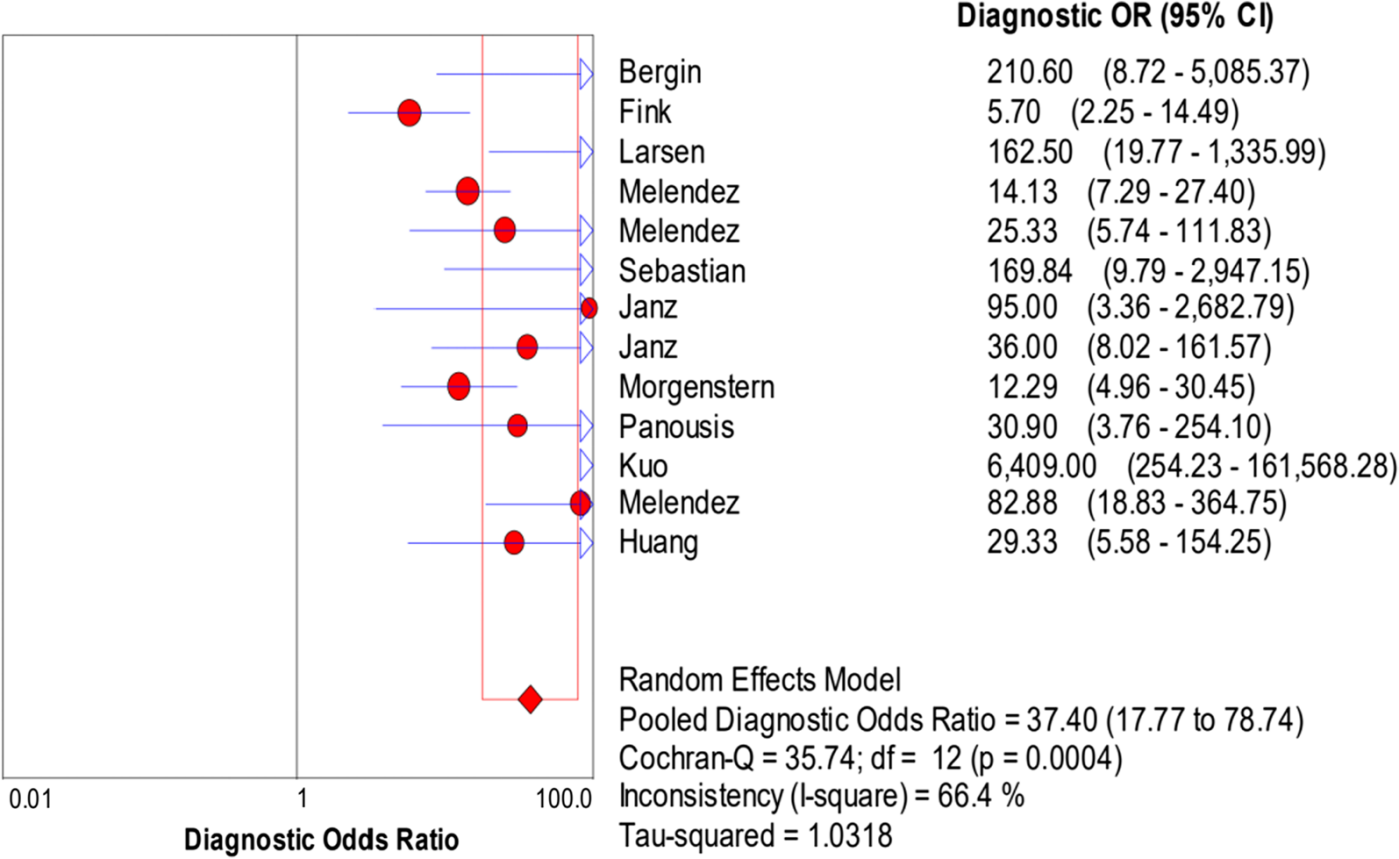
Forest plots of diagnostic odds ratio of synovial fluid PCR for PJI diagnosis

**Fig. 8:**
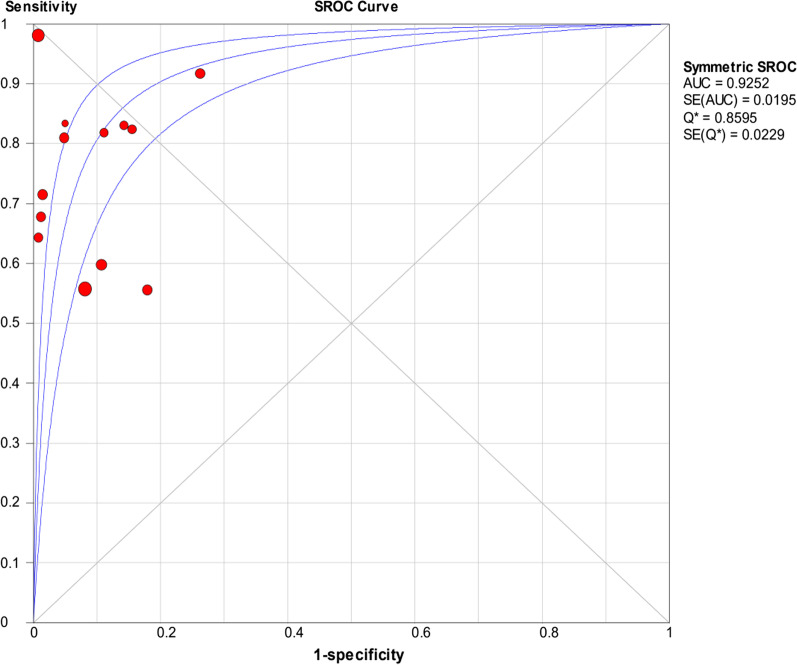
Summary of SROC of synovial fluid PCR for PJI diagnosis

The random-effects model was used to examine study heterogeneity, which was found for the sensitivity (*I*^2^ = 73.9%), specificity (*I*^2^ = 86.0%), positive likelihood ratio (PLR) (*I*^2^ = 77.4%), negative likelihood ratio (NLR) (*I*^2^ = 62.2%), and diagnostic odds ratio (DOR) (*I*^2^ = 66.4%). The pooled sensitivity, specificity, PLR, NLR, and DOR estimates for the diagnosis of PJI using synovial fluid PCR were 0.70 (95% CI 0.66–0.74), 0.92 (95% CI 0.90–0.93), 9.09 (95% CI 5.28–15.67), 0.32 (95% CI 0.24–0.42), and 37.4 (95% CI 17.77–78.74), respectively (Figs. 3, 4, 5, 6, 7). The summary receiver operating characteristic (SROC) plot showed the summary sensitivity and specificity and the 95% confidence and prediction regions, with an area under the curve (AUC) of 0.9252 (standard error of 0.0195; Fig. 8).

Synovial fluid PCR was shown to have a better sensitivity and specificity than preoperative examination of CRP and erythrocyte sedimentation rate (ESR). Similar sensitivities were observed for conventional synovial fluid culture (70%) and PCR (69%). However, the specificity of synovial fluid PCR was lower than that of synovial fluid culture (91% vs*.* 98%, respectively). Intraoperative histology, periprosthetic tissue culture, and sonicate fluid demonstrated better sensitivity and specificity than synovial fluid PCR. Details of the diagnostic values of synovial fluid PCR and other preoperative and intraoperative examination are shown in Table 2.

**Table 2:** Comparison of synovial fluid PCR with conventional diagnostic methods from the included studies

Diagnostic method	Number of studies	Sensitivity (95% CI)	Specificity (95% CI)	PLR (95% CI)	NLR (95% CI)	DOR (95% CI)	SROC (SE)
CRP	4	0.67 (0.54–0.78)	0.79 (0.74–0.83)	2.99 (2.01–4.45)	0.49 (0.26–0.92)	7.55 (3.58–15.94)	0.7969 (0.0428)
Synovial fluid PCR	4	0.79 (0.67–0.87)	0.91 (0.88–0.93)	12.05 (2.83–51.30)	0.20 (0.05–0.89)	93.69 (5.10–1721.56)	0.9613 (0.0427)
ESR	3	0.80 (0.67–0.90)	0.68 (0.63–0.73)	2.58 (2.10–3.16)	3.30 (0.17–0.52)	10.16 (4.72–21.85)	0.8284 (0.0391)
Synovial fluid PCR	3	0.93 (0.81–0.99)	0.93 (0.90–0.96)	29.92 (0.53–1678.60)	0.12 (0.01–0.93)	291.64 (12.63–6735.22)	0.9838 (0.0205)
Synovial fluid culture	9	0.70 (0.65–0.75)	0.98 (0.96–0.99)	27.26 (16.94–43.85)	0.28 (0.20–0.40)	115.11 (61.80–214.41)	0.9668 (0.0079)
Synovial fluid PCR	10	0.69 (0.64–0.74)	0.91 (0.89–0.93)	8.21 (4.56–14.77)	0.34 (0.25–0.46)	32.23 (13.94–74.50)	0.9173 (0.0239)
Tissue culture	5	0.70 (0.63–0.75)	0.92 (0.89–0.95)	13.10 (5.51–31.10)	0.30 (0.16–0.54)	41.53 (22.40–76.97)	0.9305 (0.0152)
Synovial fluid PCR	6	0.67 (0.61–0.72)	0.88 (0.85–0.91)	5.45 (3.42–8.68)	0.35 (0.26–0.48)	16.93 (10.65–26.90)	0.8740 (0.0187)
Histology	2	0.75 (0.63–0.85)	1.00 (0.96–1.00)	66.59 (9.47–468.05)	0.22 (0.07–0.69)	325.27 (24.60–4300.37)	NA
Synovial fluid PCR	2	0.64 (0.53–0.74)	0.81 (0.73–0.87)	4.17 (2.28–7.62)	0.29 (0.06–1.29)	14.20 (6.17–32.66)	NA
Sonicate fluid culture	3	0.75 (0.67–0.82)	0.96 (0.90–0.99)	11.97 (2.89–49.52)	0.26 (0.13–0.53)	50.95 (9.26–280.39)	0.9397 (0.0371)
Synovial fluid PCR	4	0.65 (0.59–0.71)	0.90 (0.86–0.93)	6.06 (4.29–8.57)	0.37 (0.26–0.53)	15.37 (9.49–24.87)	0.8662 (0.0203)

CI, confidential interval; CRP, C-reactive protein; ESR, erythrocyte sedimentation rate; PCR, polymerase chain reaction; PLR, positive likelihood ratio; NA, not available; NLR, negative likelihood ratio; DOR, diagnostic odds ratio; SROC, Summary receiver operating characteristic; SE, standard error

### Meta-regression analysis

Meta-regression analysis was performed in the group of diagnostic standards, acquisition time, number of patients, antibiotic use, and target gene (Table 3). Results showed that the most likely sources of heterogeneity were acquisition time and antibiotic treatment (*P* < 0.001). Compared with the diagnostic standards of MSIS, International Consensus Meeting (ICM), or European Bone and Joint Infection Society (EBJIS), other diagnostic criteria had a higher sensitivity of 0.85 (95% CI 0.72–0.98; *P* < 0.05). A small sample size (< 100) was observed to have a higher sensitivity of 0.81 (95% CI 0.71–0.90) compared to studies including more than 100 patients with a sensitivity of 0.71 (95% CI 0.59–0.83) (*P* = 0.01).

**Table 3:** Subgroup analysis of synovial fluid PCR

Variable (*P* value)	Number of studies	Sensitivity (95% CI)		Specificity (95% CI)	
		Adjusted	*P* value	Adjusted	*P* value
Reference standard (*P* = 0.41)					
Diagnostic standard	9	0.73 [0.64–0.83]	*P* = 0.04	0.95 [0.91–0.99]	*P* = 0.91
Others	4	0.85 [0.72–0.98]		0.94 [0.85–1.00]	
Acquisition time (*P* < 0.001)					
Preoperative	9	0.75 [0.63–0.86]	*P* = 0.57	0.96 [0.91–1.00]	*P* = 0.26
Intraoperative	2	0.77 [0.54–0.99]		0.93 [0.77–1.00]	
Number of patients (*P* = 0.34)					
Number of patients (> 100)	6	0.71 [0.59–0.83]	*P* = 0.01	0.95 [0.91–1.00]	*P* = 0.72
Number of patients (< 100)	7	0.81 [0.71–0.90]		0.93 [0.87–1.00]	
Antibiotic treatment (*P* < 0.001)					
Antibiotic treatment (yes)	5	0.73 [0.56–0.89]	*P* = 0.21	0.96 [0.91–1.00]	*P* = 0.76
Antibiotic treatment (no)	5	0.80 [0.66–0.94]		0.91 [0.82–1.00]	
Target gene (*P* = 0.1)					
16S rRNA	4	0.75 [0.59–0.90]	*P* = 0.22	0.99 [0.96–1.00]	*P* = 0.14
Others	9	0.77 [0.67–0.88]		0.92 [0.86–0.97]	

### Assessment of publication bias

**Fig. 9:**
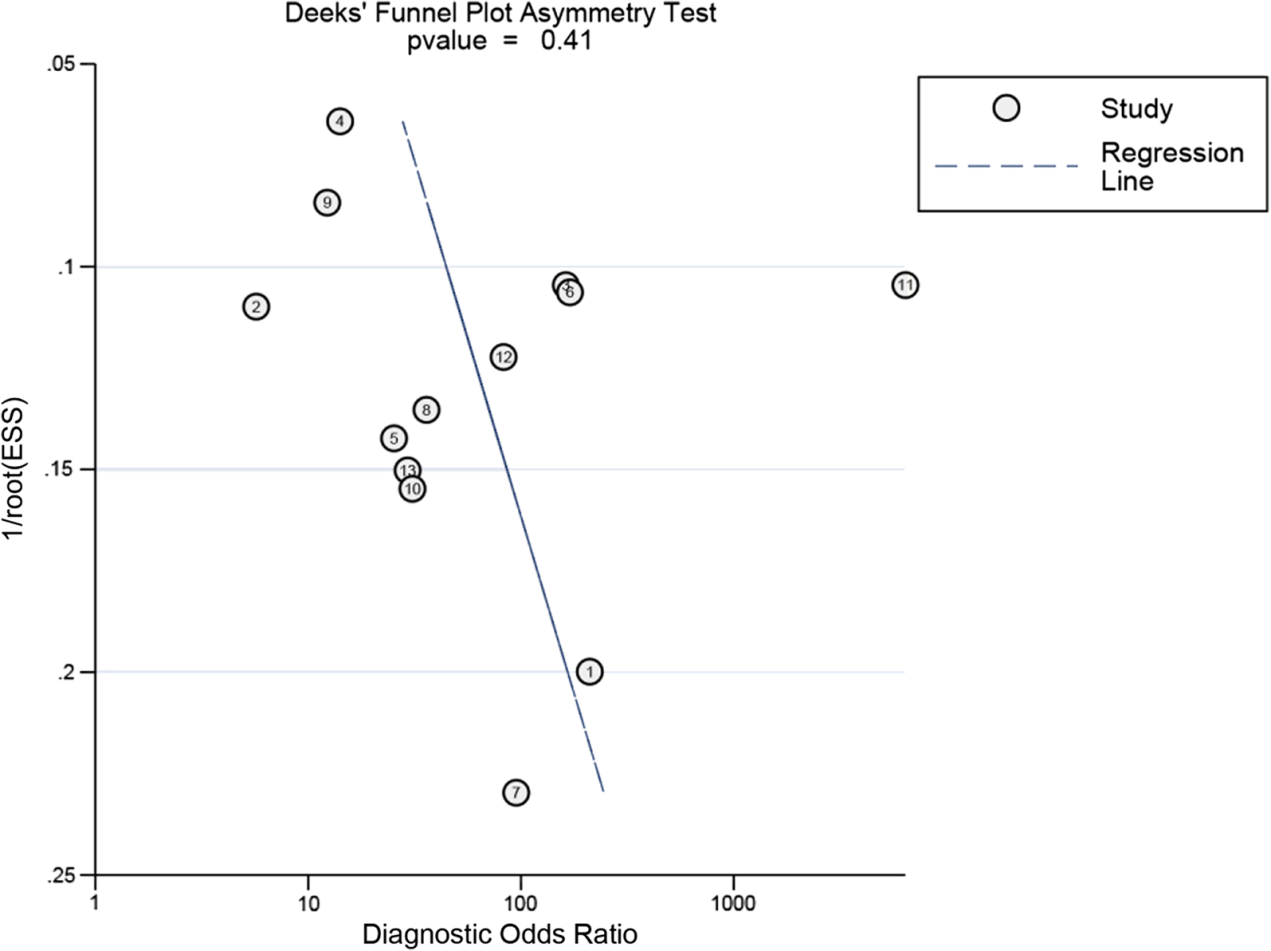
Deeks’ funnel plot for evaluation of publication bias

Deeks’ funnel plot analysis did not identify a potential publication bias for synovial fluid PCR (*P* = 0.41; Fig. 9).

## Discussion

The present meta-analysis showed that the pooled sensitivity and specificity of synovial fluid PCR are 70% and 92%, respectively. The AUC value of the SROC was 0.9252. These results suggest that synovial fluid PCR could be used for the diagnosis of infection after hip and knee arthroplasty. The first meta-analysis of the use of PCR in the diagnosis of PJI presented the sensitivity and specificity of synovial fluid (84% and 89%, respectively), tissue samples (95% and 81%, respectively), and sonicate fluid (81% and 96%, respectively) [16], with moderate sensitivity and specificity levels observed for the three sample types. Interestingly, similar results were also reported by the retrospective study performed by Huang and colleagues [17]. In 2018, Jun et al. [15] performed a diagnostic meta-analysis of PCR in PJI, reporting a sensitivity of 0.76 (95% CI 0.65–0.85) and specificity of 0.94 (95% CI 0.92–0.95). Unfortunately, the pooled result combines samples from synovial fluid, sonicate fluid, and intraoperative tissue. Hence, the diagnostic value of synovial fluid PCR in periprosthetic hip and knee by meta-analysis remained unexplored. The present study is the first meta-analysis evaluating the diagnostic value of synovial fluid PCR for diagnosing infection after hip and knee replacement.

In recent years, the use of joint fluid for diagnosing PJI was a topic of considerable interest. Multiple synovial fluid tests were applied in clinical practice, with several valuable tests incorporated into the new definition of MSIS [10]. Through a literature review of the published meta-analysis of these synovial fluid methods from the MSIS guideline [26–30], the pooled sensitivity of these tests is superior to that of synovial fluid PCR. The specificity of synovial fluid PCR exceeded that of synovial fluid interleukin-6 (IL-6), CRP, WBC, and PMN, but was lower than synovial fluid culture, alpha-defensins, and LE. Based on the AUC value of meta-analysis of synovial fluid [31], only LE demonstrated excellent accuracy in the diagnosis of PJI, followed by alpha-defensins, IL-6, CRP, and PMN. Furthermore, synovial fluid WBC as well as PCR showed good accuracy (Table 4). Although the results of most synovial fluid biomarkers were superior to that of the synovial fluid PCR [26–30], synovial fluid culture and some PCR tests can detect bacteria and could provide a more valuable reference for further comparisons with intraoperative diagnostic results. However, the diagnostic value between synovial fluid culture and PCR for PJI detection is controversial.

**Table 4:** Comparison of synovial fluid PCR with synovial fluid biomarkers using the MSIS definition based meta-analysis results

References	Test	Location	Number of studies	Sensitivity (95% CI)	Specificity (95% CI)	DOR (95% CI)	AUC
[26]	Synovial fluid alpha-defensin	Hip, knee, shoulder	16	0.87 (0.83–0.90)	0.97 (0.96–0.98)	158.18 (74.26–336.91)	0.9685
[26]	Synovial fluid LE	Hip, knee, shoulder	12	0.87 (0.84–0.90)	0.96 (0.95–0.97)	170.09 (97.63–296.32)	0.9818
[27]	Synovial fluid IL-6	Hip, knee, shoulder	8	0.91 (0.82–0.96)	0.90 (0.84–0.95)	101 (28–358)	0.96
[28]	Synovial fluid CRP	Hip, knee	6	0.92 (0.86–0.96)	0.90 (0.87–0.93)	101.40 (48.07–213.93)	0.9663
[29]	Synovial fluid WBC count	Hip, knee	11	0.900 (0.872–0.922)	0.898 (0.814–0.947)	84.4 (40.3–157)	0.910
[29]	Synovial fluid PMN	Hip, knee	11	0.906 (0.870–0.933)	0.861 (0.808–0.901)	62.8 (32.7–109)	0.940
[30]	Synovial fluid culture	Hip, knee	34	0.72 (0.65–0.78)	0.95 (0.93–0.97)	52 (31–86)	0.94
Current study	Synovial fluid PCR	Hip, knee	11	0.70 (0.66–0.74)	0.92 (0.90–0.93)	37.4 (17.77–78.74)	0.9252

CI, confidence interval; AUC, Area under the curve; DOR, Diagnostic odds ratio; CRP, C-reactive protein; IL-6, Interleukin-6; LE, Leukocyte esterase; PCR, Polymerase chain reaction; PMN, Polymorphonuclear leukocytes; WBC, White blood cell

Synovial fluid PCR was compared with preoperative and intraoperative tests from the included studies The PCR test was found to have better sensitivity and specificity than that of serum CRP and ESR. In contrast, lower sensitivity and specificity were observed in comparison to all intraoperative methods. Compared with synovial fluid culture, synovial fluid PCR had an almost identical level of sensitivity with synovial fluid culture (69% vs*.* 70%, respectively) and a lower specificity level (91% vs. 98%, respectively). Synovial fluid PCR and culture were also observed to have similar results in comparison to the previous meta-analysis of synovial fluid culture, with a sensitivity of 70% and 72%, respectively, and a specificity of 92% and 95%, respectively [30]. Although the current meta-analysis and our subgroup results showed that the sensitivity and specificity of synovial fluid PCR were lower than that of synovial fluid, PCR has several advantages in regard to the detection of bacteria. Synovial fluid PCR has been reported to rapidly provide results within 3–72 h [14, 23, 25], and could also detect culture-negative bacteria [11, 12, 21, 22, 25]. Sujeesh and co-workers reported that the sensitivity of 16S rRNA PCR and synovial fluid culture was 68.1% and 70.2%, respectively [22]. PJI was detected by PCR in five cases that were negative by synovial fluid culture. Synovial fluid multiplex PCR identified 12 cases negative by synovial fluid culture, with 10 cases caused by low-virulence bacteria (coagulase-negative staphylococci and *Cutibacterium acnes*) [25]. The author also found that more cases of polymicrobial infections were detected by synovial PCR than synovial fluid culture (four *vs.* two cases), with similar results also reported by Melendez and colleagues [18, 21]. Due to rare cases of mixed infection in these studies, further research is required. However, in cases treated with antibiotics before specimen collection, the testability of synovial fluid PCR was lower than synovial fluid culture. A comparison of the PCR panel and synovial fluid culture in patients that received antibiotics within 30 days before joint aspiration revealed the sensitivity of the PCR panel and synovial culture to be 64.5% and 85.4%, respectively [21]. In another study, PCR-ESI/MS detected eight of nine PJI cases who had received antibiotics within 30 days, whereas synovial fluid culture detected all nine cases [18]. The use of antibiotics before PCR analysis most likely impacts culture results. Meta-regression analysis results from the current study show that the sensitivity level of cases receiving antibiotic therapy were less than cases without antibiotic therapy (73% vs. 80%). Moreover, meta-regression was also analyzed in the preoperative and intraoperative aspiration groups, with slightly higher sensitivity and lower specificity observed for the intraoperative test compared to the preoperative test (sensitivity: 77% vs. 75%, respectively; specificity: 93% vs. 96%). In contrast, the meta-analysis of synovial fluid white cell count performed by Qu and co-workers [32] found that preoperative collection had a higher sensitivity than intraoperative samples (91% vs. 77%, respectively), and lower specificity than that of intraoperative samples (89% vs. 97%). However, due to the limited data of the studies included from our intraoperative study (two studies) and different tests performed in these two meta-analyses, whether intraoperative and preoperative sample collection infers with the diagnostic accuracy remains an avenue for further exploration.

Although various types of synovial fluid PCR have been tested in the clinical diagnosis of PJI, the diagnostic ability has most likely been disregarded. The most frequently described disadvantage of PCR is FP results, with the potential impacting factors, including the use of different target genes, PCR type, laboratory technician skills, and laboratory conditions [13, 19, 22, 24]. The use of 16S/28S rRNA RT-PCR with high-quality control standards demonstrated excellent results, with a sensitivity of 100% and specificity of 99.5% [13].Sebastian and colleagues [22] found that DNase treatment could reduce exogenous bacterial contamination, with no FP results observed in synovial fluid PCR: however, the sensitivity was affected. Further studies are required to determine the most suitable type of PCR for PJI diagnosis and the standard procedure required.

The present study has several limitations. First, the identified studies used different types of PCR; therefore, the overall result may impact the estimates of diagnostic accuracy. Second, meta-regression analysis was not performed in regard to the prosthesis type or sample condition to further explore sources of heterogeneity. The type of prosthesis described in the included studies were the knee or both the hip and knee; however, studies focusing only on the periprosthetic hip were not found. Therefore, further analysis of differences between the hip and knee could not be performed in meta-regression analysis. Regarding the sample condition, frozen specimens were used in the studies; however, there was ambiguity in terms of the use of fresh samples. Third, the diagnostic accuracy of synovial fluid PCR may be affected by the standard definition of PJI [33]. Fourth, the current meta-analysis only included the English article, which was published in the database of Web of Science, PubMed, and Scopus. However, the relevant published literature from other languages or other databases is probably lacking.

## Conclusions

The diagnostic capability of synovial fluid PCR is not superior to that of synovial fluid culture. However, in cases of negative synovial fluid culture with highly suspected early-stage infection, synovial fluid PCR can be used as a rapid diagnostic confirmatory tool.

## Data Availability

Not applicable.

## Abbreviations

AUC: Area under the curve
C: Clinical signs of infection
CI: Confidence interval
CRP: C-reactive protein
DOR: Diagnostic odds ratio
EBJIS: European Bone and Joint Infection Society
ESI/MS: Electrospray ionization mass spectrometry
ESR: Erythrocyte sedimentation rate
FN: False-negative
FP: False-positive
H: Histological examination
IDSA: Infectious Diseases Society of America
ICM: International Consensus Meeting
IL-6: Interleukin-6
LE: Leukocyte esterase
M: Microbiological or laboratory examination
MeSH: Medical subject headings
MSIS: Musculoskeletal Infection Society
NLR: Negative likelihood ratio
N: No
NA: Not available
P: Presence sinus tract or purulence around the prosthesis
PCR: Polymerase chain reaction
PJI: Periprosthetic joint infection
PLR: Positive likelihood ratio
PMN: Polymorphonuclear
QUADAS-2: Quality Assessment of Diagnostic Accuracy Studies-2
qPCR: Quantitative PCR
R: Radiographic images
RT: Real-time
SROC: Summary receiver operating characteristic
SE: Standard error
Sen: Sensitivity
Spe: Specificity
TN: True negative
TP: True positive
WBC: White blood cell count
Y: Yes
